# Isoform-Specific Transcriptomic Effects of *miR-133A1*, *miR-133A2*, and *miR-133B* in a Colorectal Cancer Cell Line

**DOI:** 10.3390/genes16111322

**Published:** 2025-11-03

**Authors:** Ji Su Mo, Youn Ho Han

**Affiliations:** 1 Digestive Disease Research Institute, Wonkwang University, Iksan 54538, Republic of Korea; 2 Department of Oral Pharmacology, College of Dentistry, Wonkwang University, Iksan 54538, Republic of Korea

**Keywords:** *miR-133A1*, *miR-133A2*, *miR-133B*, colorectal cancer, transcriptomic profiling

## Abstract

Background: MicroRNA-133 (*miR-133*) has been implicated in diverse cancers as a tumor suppressor, yet the isoform-specific contributions of *miR-133A1*, *miR-133A2*, and *miR-133B* in colorectal cancer (CRC) remain unclear. Methods: We established stable colorectal cancer cell lines expressing each *miR-133* isoform and performed isoform-level transcriptomic profiling. Differentially expressed genes (DEGs) were identified relative to parental cells and subjected to gene ontology (GO) and KEGG enrichment analyses. Comparative analyses highlighted both shared and distinct biological pathways regulated by each isoform. Results: Venn diagram and clustering analyses revealed that all three isoforms shared a core regulatory program, with 34 genes consistently upregulated and 195 genes downregulated across all isoforms, while also displaying isoform-specific DEGs. *miR-133A1*, *miR-133A2*, and *miR-133B* showed predominantly convergent transcriptional programs, with subtle quantitative differences observed primarily in KI*133B*. Heatmap analysis of representative genes confirmed both overlapping and isoform-specific expression changes, with survival- and proliferation-associated genes more strongly upregulated in *miR-133A2* and *miR-133B*. Conclusion: These findings suggest that *miR-133* isoforms exert both shared and subtly divergent regulatory functions in colorectal cancer, coordinating apoptosis, proliferation, migration, and signaling network modulation. Isoform-specific transcriptional regulation of *miR-133* may contribute to tumor progression and represents a potential biomarker and therapeutic target in CRC.

## 1. Introduction

Colorectal cancer (CRC) is among the most common malignancies worldwide and remains a leading cause of cancer-related mortality [1,2]. The disease arises through a multistep process involving genetic mutations and epigenetic dysregulation, with well-established hallmarks, including activation of the WNT/β-catenin pathway, mutations in *KRAS*, loss of *TP53*, and aberrant regulation of apoptosis and proliferation [3,4,5]. Despite advances in targeted therapy and precision oncology, prognosis for advanced CRC remains poor [6], highlighting the need to identify novel molecular regulators and therapeutic strategies.

MicroRNAs (miRNAs) are small non-coding RNAs that regulate gene expression post-transcriptionally and play pivotal roles in cancer biology [7,8]. Among them, *miR-133A* is generally considered a tumor suppressor, with documented functions in regulating apoptosis, proliferation, and metastasis [9,10]. Dysregulation of *miR-133A* has been reported in several cancers, but most studies have examined it as a single entity, without distinguishing between its isoforms [11,12]. *miR-133* family members include *miR-133A1* and *miR-133A2*, which originate from separate genomic loci but produce identical mature sequences, and *miR-133B*, which differs by a single nucleotide and lacks a co-encoded passenger strand from the same precursor [13,14]. These distinctions may influence strand selection, processing efficiency, and transcriptome-wide effects.

Isoform switching in microRNAs is increasingly recognized as a critical regulatory mechanism in cancer biology [15,16]. In colorectal cancer, miRNA isoform variation has been implicated in regulating key oncogenic pathways such as *KRAS* and *TP53*, highlighting the potential role of isoform-specific expression patterns in tumor heterogeneity, progression, and therapy resistance [17,18]. Therefore, isoform-level characterization is essential to fully understand the biological functions of miRNAs in tumorigenesis. In CRC, the contribution of miR-133 isoforms has not been systematically explored.

The present study sought to characterize the isoform-specific transcriptomic effects of *miR-133* in CRC. By integrating differential expression, functional enrichment, and comparative analyses across stable cell lines expressing each isoform, we aimed to uncover both shared and divergent regulatory programs of *miR-133A1*, *miR-133A2*, and *miR-133B.* Our results reveal isoform-specific modulation of key oncogenic pathways, providing novel insights into CRC pathogenesis and highlighting potential avenues for biomarker and therapeutic development.

## 2. Materials and Methods

### 2.1. Cell Culture and Establishment of Stable Cell Lines

Human colorectal cancer SW48 cells were obtained from the American Type Culture Collection (ATCC, Manassas, VA, USA; Cat. No. CCL-231) and maintained in RPMI-1640 medium (Gibco) supplemented with 10% fetal bovine serum (FBS; HyClone, Logan, UT, USA), 100 U/mL penicillin, and 100 µg/mL streptomycin at 37 °C in a humidified atmosphere with 5% CO_2_. Stable cell lines overexpressing *miR-133A1* (SW48-KI133A1), *miR-133A2* (SW48-KI133A2), and *miR-133B* (SW48-KI133B) were generated using the pmRi-ZsGreen1 vector (Takara Bio Inc., Shiga, Japan) carrying each isoform under the control of a CMV promoter. Precursor sequences of *miR-133A1*, *miR-133A2*, and *miR-133B* were amplified and cloned into the pmRi-ZsGreen1 vector (Takara, Cat. No. 631121) using standard restriction sites. Constructs were confirmed by Sanger sequencing. Stable SW48 cell lines were established under doxycycline induction (0.5 μg/mL). Cells transduced with an empty pmRi-ZsGreen1 vector were used as negative controls.

### 2.2. RNA Extraction and Quality Assessment

Total RNA was isolated from SW48 parental and stable isoform-expressing cells using TRIzol reagent (Invitrogen, Carlsbad, CA, USA) according to the manufacturer’s instructions. RNA concentration and purity were determined by NanoDrop spectrophotometry (Thermo Fisher Scientific, Waltham, MA, USA), and integrity was confirmed using the Agilent 2100 Bioanalyzer (Agilent Technologies, Santa Clara, CA, USA). Samples with RNA integrity number (RIN) > 8.0 were used for sequencing.

### 2.3. Quantitative Real-Time PCR (qRT-PCR) for MicroRNA Expression Validation

Functional validation of stable cell lines was performed to confirm robust overexpression of each mature miRNA compared with vector controls. For mature microRNA quantification, complementary DNA (cDNA) was synthesized from 1 μg of total RNA using the TB Green miRNA RT Reagent Kit (Takara, Cat. No. RR716A) according to the manufacturer’s protocol. The reverse transcription reaction was performed at 42 °C for 60 min, followed by enzyme inactivation at 95 °C for 5 min. Quantitative PCR was performed using the TB Green Premix Ex Taq II (Takara, Cat. No. RR820A) with miRNA-specific forward primers and the universal reverse primer provided in the RT kit. PCR reactions were carried out in 20 μL volumes containing 10 μL of 2× TB Green Premix Ex Taq II (Takara Bio Inc., Shiga, Japan), 0.5 μL each of forward and reverse primers (10 μM), 2 μL of diluted cDNA template, and 7 μL of nuclease-free water. Thermal cycling was performed on a 7500 Real-Time PCR System (Applied Biosystems, Foster City, CA, USA) with the following conditions: initial denaturation at 95 °C for 30 s, followed by 40 cycles of denaturation at 95 °C for 5 s and annealing/extension at 60 °C for 20 s. Expression levels were normalized to U6 small nuclear RNA (snRNA) as an internal control. Relative expression was calculated using the 2^(−ΔΔCt) method, with empty vector control samples set as calibrator (fold change = 1). All experiments were performed in triplicate with three independent biological replicates to ensure reproducibility and statistical validity.

### 2.4. RNA Sequencing and Data Processing

High-throughput RNA sequencing (RNA-seq) was performed on the Illumina NovaSeq 6000 platform (Illumina, San Diego, CA, USA) to generate 150 bp paired-end reads. Two biological replicates were prepared for each condition, including parental SW48 cells and each of the three *miR-133* isoform knock-in cell lines (SW48-KI*133-A1*, SW48-KI*133*-*A2*, and SW48-KI*133-B*). Sequencing libraries were prepared using the NEBNext Ultra II RNA Library Prep Kit (New England Biolabs, Ipswich, MA, USA), and sequencing was performed to achieve an average depth of approximately 40–50 million paired-end reads per sample. Raw reads were quality-checked with FastQC v0.11.9 (Babraham Bioinformatics, Cambridge, UK), and adapter sequences and low-quality bases were trimmed using Trimmomatic v0.39 (Usadel Lab, Aachen, Germany). Clean reads were aligned to the human reference genome (GRCh38/hg38) with STAR aligner v2.7.10a (Cold Spring Harbor Laboratory, Cold Spring Harbor, NY, USA). Gene- and isoform-level transcript quantification was performed using Salmon v1.10.0 (Patro Lab, University of Virginia, Charlottesville, VA, USA) and Kallisto v0.48.0 (Pachter Lab, California Institute of Technology, Pasadena, CA, USA).

### 2.5. Differential Expression Analysis

Differential expression analysis was conducted using DESeq2 v1.36.0 (Bioconductor, R Foundation, Vienna, Austria) in R v4.2.2 (R Foundation for Statistical Computing, Vienna, Austria). Genes with adjusted *p*-value < 0.05 and |log_2_ fold change| ≥ 1 were considered significantly differentially expressed. Isoform-level differential usage and potential isoform switching events were assessed with IsoformSwitchAnalyzeR v1.18.0 (Bioconductor, University of Copenhagen, Copenhagen, Denmark). All analyses were performed using at least two independent biological replicates.

### 2.6. Gene Ontology and Pathway Enrichment Analysis

Significantly up- and down-regulated genes from each isoform were subjected to Gene Ontology (GO) and Kyoto Encyclopedia of Genes and Genomes (KEGG) pathway enrichment analyses using the clusterProfiler v4.4.4 (Bioconductor, Southern Medical University, Guangzhou, China) R package. Enrichment significance was defined as false discovery rate (FDR, Benjamini–Hochberg correction) < 0.05. Results were visualized as bar plots, bubble plots, and heatmaps using ggplot2 v3.4.2 (RStudio, Boston, MA, USA) and pheatmap v1.0.12 (CRAN, R Foundation, Vienna, Austria).

### 2.7. Clustering and Visualization of Differentially Expressed Genes

Unsupervised hierarchical clustering and heatmap visualization of differentially expressed genes were performed using the pheatmap v1.0.12 (CRAN, R Foundation, Vienna, Austria) package in R. Log_2_-transformed normalized counts were used to assess similarities across replicates and isoforms. Venn diagrams were generated using VennDiagram v1.7.3 (Bioconductor, University Health Network, Toronto, ON, Canada), and volcano plots were produced using EnhancedVolcano v1.16.0 (Bioconductor, University of Cambridge, Cambridge, UK) to highlight overlaps and isoform-specific expression profiles.

### 2.8. Statistical Analyses

All experiments were conducted with at least three independent biological replicates. RNA-seq differential expression analysis was performed using DESeq2 v1.36.0 with Benjamini–Hochberg correction for false discovery rate (FDR). For qRT-PCR validation, statistical significance was assessed using two-tailed Student’s *t*-test (two groups) or one-way ANOVA with Tukey’s post hoc test (multiple groups). Data are expressed as mean ± standard deviation (SD), and significance was defined as *p* < 0.05. Exact replicate numbers and statistical tests applied are described in each figure legend.

## 3. Results

### 3.1. Sequence Characteristics and Differential Functional Pathways Regulated by miR-133 Isoforms

**Figure 1:**
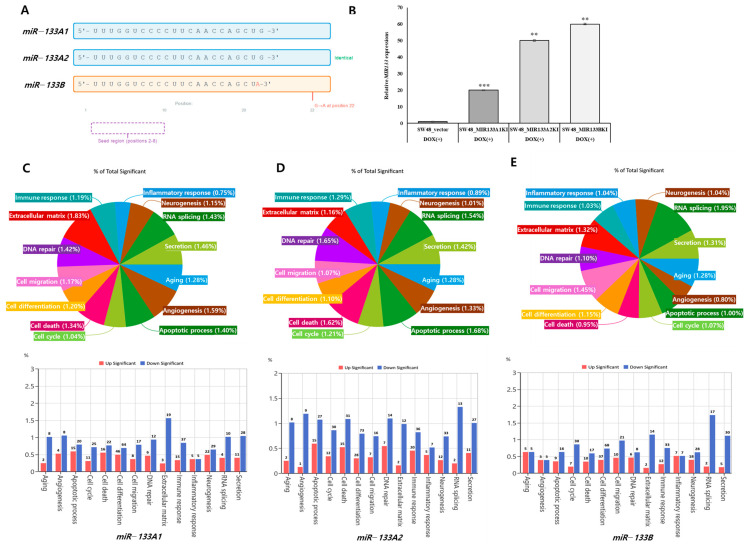
Functional categorization of differentially expressed genes regulated by *miR-133* isoforms. (**A**) Schematic representation of mature *miR-133* isoform sequences. *miR-133A1* and *miR-133A2* are identical, while *miR-133B* contains a G→A substitution at position 22. The seed region (positions 2–8, dashed purple box) is identical across all isoforms. (**B**) qRT-PCR validation of *miR-133* overexpression in SW48 cells. Data represent mean ± SD (n = 3). (** *p* < 0.01, *** *p* < 0.001 vs. vector control). DOX(−): non-induced; DOX(+): doxycycline-induced. (C-E) Functional categorization of differentially expressed genes in *miR-133A1* (**C**), *miR-133A2* (**D**), and *miR-133B* (**E**) stable cell lines. Pie charts show proportional distribution across biological processes; bar graphs show directional regulation (red: upregulated; blue: downregulated).

The *miR-133* family consists of three isoforms with highly similar sequences. *miR-133A1* and *miR-133A2* produce identical mature sequences (5′-UUUGGUCCCCUUCAACCAGCUG-3′), while *miR-133B* differs by a single nucleotide at position 22 (G→A) (Figure 1A). Despite sharing an identical seed region (positions 2–8), transcriptomic profiling revealed both shared functions and subtle quantitative divergence among these isoforms. We established stable overexpression of each isoform in SW48 colorectal cancer cells and validated expression levels by qRT-PCR. All three microRNAs showed robust overexpression: *miR-133A1* (20-fold, *p* < 0.001), *miR-133A2* (50-fold, *p* < 0.01), and *miR-133B* (60-fold, *p* < 0.01) compared to vector control (Figure 1B). Although expression magnitudes differed, the overall direction and category of transcriptomic changes were consistent across isoforms, suggesting that these effects are not merely dosage dependent but reflect distinct regulatory mechanisms. Transcriptomic analysis revealed distinct regulatory patterns for each isoform. *miR-133A1*-expressing cells showed enrichment in extracellular matrix regulation (1.83%), cell migration (1.17%), and apoptotic processes (1.40%) (Figure 1C). *miR-133A2* preferentially upregulated RNA splicing (1.54%) and immune response pathways (1.29%) while downregulating apoptosis (Figure 1D). In contrast, *miR-133B* enhanced RNA processing (1.95%) and secretion (1.31%) but suppressed immune-related functions (1.03%) (Figure 1E). Notably, inflammatory response genes showed divergent regulation: upregulation in *miR-133A1/A2* cells but downregulation in *miR-133B* cells. These findings demonstrate that *miR-133* isoforms exert both overlapping and unique transcriptional control despite identical seed sequences.

### 3.2. Overlapping and Isoform-Specific Gene Expression Changes Induced by miR-133 Isoforms

**Figure 2:**
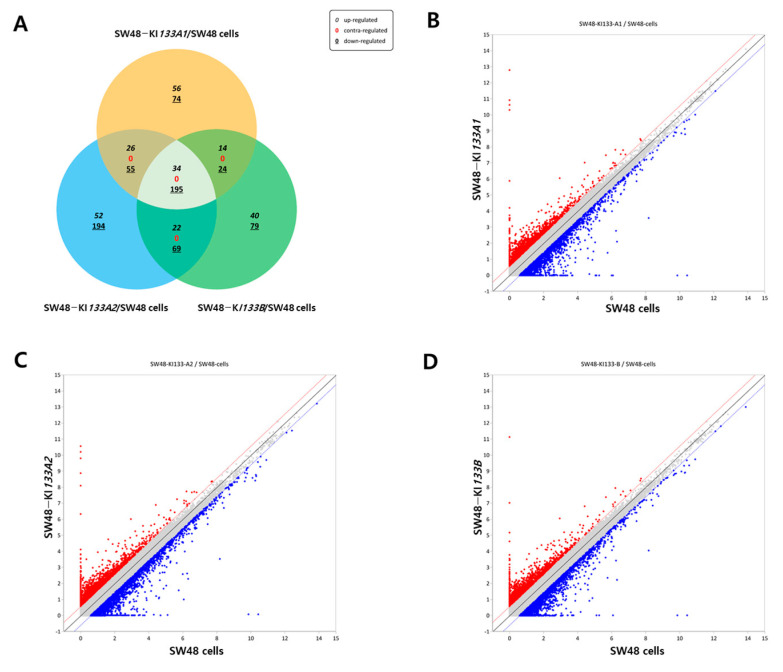
Comparative analysis of differentially expressed genes regulated by *miR-133* isoforms. (**A**) Venn diagram showing the overlap of differentially expressed genes among SW48-KI133A1, SW48-KI133A2, and SW48-KI133B cells compared with parental SW48 controls. Numbers indicate isoform-specific and shared upregulated (red) and downregulated (blue) genes. (**B**–**D**) Scatter plots depicting log2-normalized expression changes in SW48-KI133A1 (**B**), SW48-KI133A2 (**C**), and SW48-KI133B (**D**) relative to SW48 control cells. Red dots represent significantly upregulated genes, while blue dots indicate significantly downregulated genes. The results highlight both common and isoform-specific transcriptional signatures regulated by *miR-133* isoforms.

To dissect the transcriptional impact of each isoform, we compared DEGs among SW48-KI133A1, SW48-KI133A2, and SW48-KI133B relative to parental controls. Venn diagram analysis revealed a shared set of 34 commonly upregulated and 195 downregulated genes across all isoforms (Figure 2A). Each isoform also displayed unique DEGs: 74 up- and 56 down-regulated genes in KI133A1, 52 up- and 194 down-regulated in KI133A2, and 40 up- and 79 down-regulated in KI133B. Scatter plots showed the distribution of upregulated (red) and downregulated (blue) transcripts in KI133A1 (Figure 2B), KI133A2 (Figure 2C), and KI133B (Figure 2D). Comparative analysis among isoforms revealed high overall correlation (r > 0.95) between replicates, confirming reproducibility, while subtle quantitative differences in DEG distribution highlight isoform-specific effects rather than random variation. Together, these results indicate that *miR-133* isoforms share a reproducible core transcriptional program, with minor but consistent isoform-specific signatures that cannot be explained by noise or dosage differences alone.

### 3.3. Clustering of Differentially Expressed Genes in miR-133 Isoform-Expressing Cells

**Figure 3:**
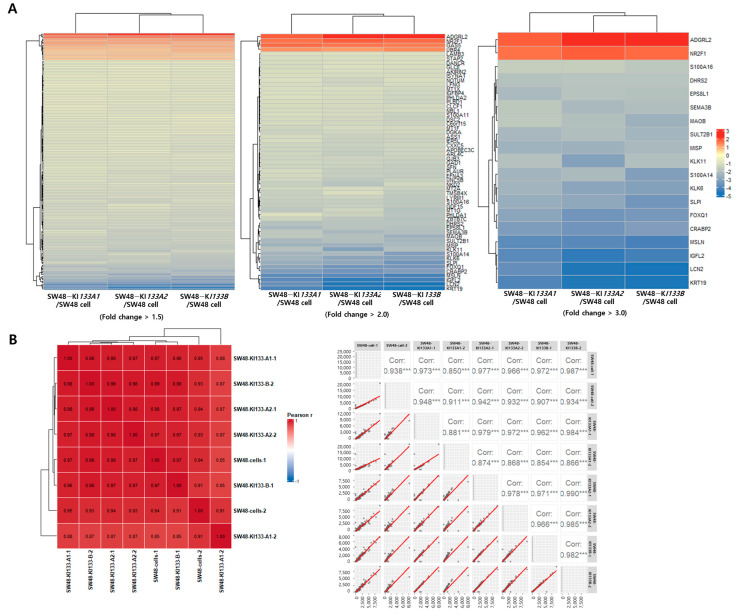
Hierarchical clustering and correlation analysis of differentially expressed genes in *miR-133* isoform-expressing SW48 cells. (**A**) Heatmaps showing hierarchical clustering of differentially expressed genes at three-fold change thresholds (>1.5, >2.0, and >3.0) for SW48-KI*133A1*, SW48-KI*133A2*, and SW48-KI*133B* cells compared with parental SW48 controls. Rows represent individual genes, and columns represent two independent biological replicates. Red indicates upregulated genes, and blue indicates downregulated genes. At higher thresholds, core isoform-specific genes (labeled on the right) are highlighted. KI*133A1* and KI*133A2* show similar expression patterns, whereas KI*133B* displays a distinct transcriptional profile. (**B**) Pearson correlation matrix (left) and scatter plots (right) of all biological replicates across samples. Strong correlations (r = 0.95–0.99) confirm high reproducibility between replicates and support the robustness of differential expression analysis. These results indicate that the observed transcriptomic differences among isoforms are biologically reproducible rather than experimental noise. Asterisks indicate statistical significance (*** *p* < 0.001).

Unsupervised hierarchical clustering analysis confirmed global transcriptomic reprogramming induced by each isoform. To systematically evaluate the robustness of differential gene expression, we applied stringent fold change thresholds (>1.5, >2.0, and >3.0) and visualized the resulting gene clusters (Figure 3A). Both biological replicates per condition were included in the heatmap, demonstrating nearly identical clustering patterns that confirm experimental reproducibility. At all threshold levels, heatmaps demonstrated clear segregation of isoform-expressing cells from parental SW48 controls. Notably, KI*133A1* and KI*133A2* clustered more closely at lower thresholds, reflecting their high transcriptional similarity, whereas KI*133B* exhibited a more distinct profile. The inclusion of replicate-level visualization provides direct evidence that the observed transcriptional divergence among isoforms is biologically consistent and not due to technical noise. As the fold change threshold increased (>3.0), the number of differentially expressed genes decreased, but core regulatory genes such as *ADGRL2*, *NR2F1*, *S100A16*, and *KRT19* remained consistently altered, highlighting isoform-specific regulation of key cancer-associated genes. Upregulated genes (red) were enriched in proliferation, cytoskeletal remodeling, and RNA processing, whereas downregulated genes (blue) were mainly involved in apoptosis and immune response. Sample correlation analysis further confirmed high reproducibility between biological replicates, with Pearson correlation coefficients ranging from 0.85 to 0.99 (Figure 3B). Strong correlations (>0.97) were observed within replicates of the same condition, while moderate correlations (0.85–0.93) between different isoforms reflected both shared and isoform-specific transcriptional programs. Together, these results demonstrate robust and reproducible transcriptomic alterations induced by *miR-133* isoforms.

### 3.4. Functional Enrichment Analysis of Genes Regulated by miR-133 Isoforms

**Figure 4:**
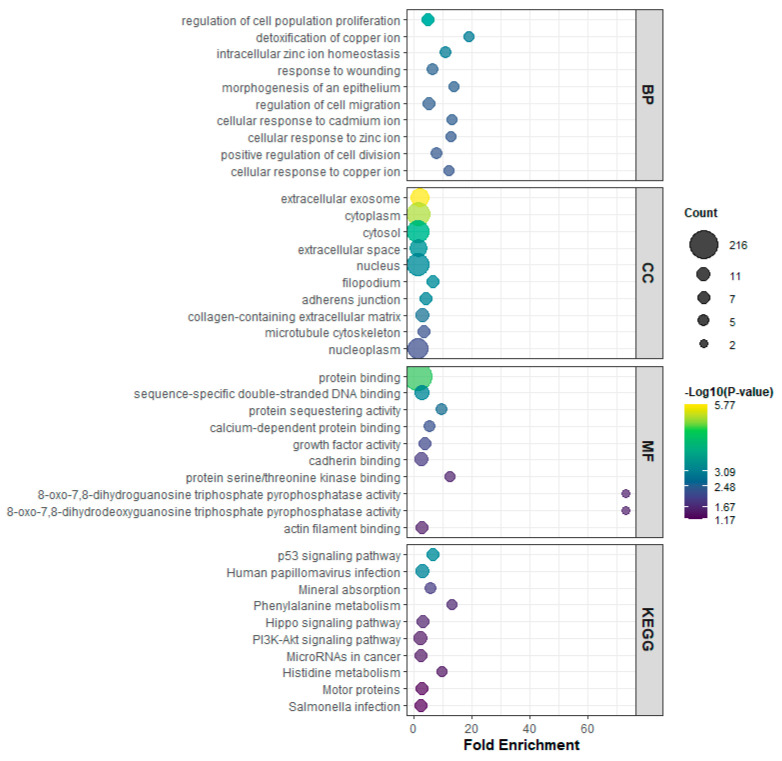
Gene ontology (GO) and KEGG pathway enrichment analysis of genes regulated by *miR-133* isoforms. The plots display enriched terms across four categories: Biological Process (BP), Cellular Component (CC), Molecular Function (MF), and KEGG pathways. The x-axis represents fold enrichment, bubble size indicates the number of genes (count) associated with each term, and the color scale reflects statistical significance (–log_10_ *p*-value). Enrichment results demonstrate that *miR-133* isoforms regulate pathways related to cell proliferation, metal ion homeostasis, cellular stress responses, extracellular matrix regulation, and major cancer-associated signaling cascades including p53, PI3K-Akt, and Hippo pathways.

GO and KEGG pathway enrichment analyses revealed that *miR-133* isoforms coordinately regulate cancer-associated biological processes. In the Biological Process category, enriched terms included regulation of cell population proliferation, detoxification of copper ion, intracellular zinc ion homeostasis, response to wounding, morphogenesis of epithelium, regulation of cell migration, and cellular responses to metal ions including cadmium, zinc, and copper (Figure 4, BP panel). Cellular Component terms highlighted extracellular exosome, cytoplasm, cytosol, extracellular space, nucleus, filopodium, adherens junction, collagen-containing extracellular matrix, microtubule cytoskeleton, and nucleoplasm (Figure 4, CC panel). Molecular Function terms were enriched for protein binding, sequence-specific double-stranded DNA binding, protein sequestering activity, calcium-dependent protein binding, growth factor activity, cadherin binding, protein serine/threonine kinase binding, 8-oxo-7,8-dihydrodeoxyguanosine triphosphate pyrophosphatase activity, 8-oxo-7,8-dihydroguanosine triphosphate pyrophosphatase activity, and actin filament binding (Figure 4, MF panel). KEGG pathway analysis further identified p53 signaling pathway, human papillomavirus infection, mineral absorption, phenylalanine metabolism, Hippo signaling pathway, PI3K-Akt signaling pathway, microRNAs in cancer, histidine metabolism, motor proteins, and Salmonella infection as significantly enriched pathways (Figure 4, KEGG panel). Taken together, these results demonstrate that *miR-133* isoforms coordinately regulate genes that control proliferation, apoptosis, migration, metal ion homeostasis, and stress responses through multiple signaling pathways, with potential implications in cancer biology and cellular stress adaptation.

### 3.5. Isoform-Specific Functional Pathway Regulation by miR-133A1, miR-133A2, and miR-133B

**Figure 5:**
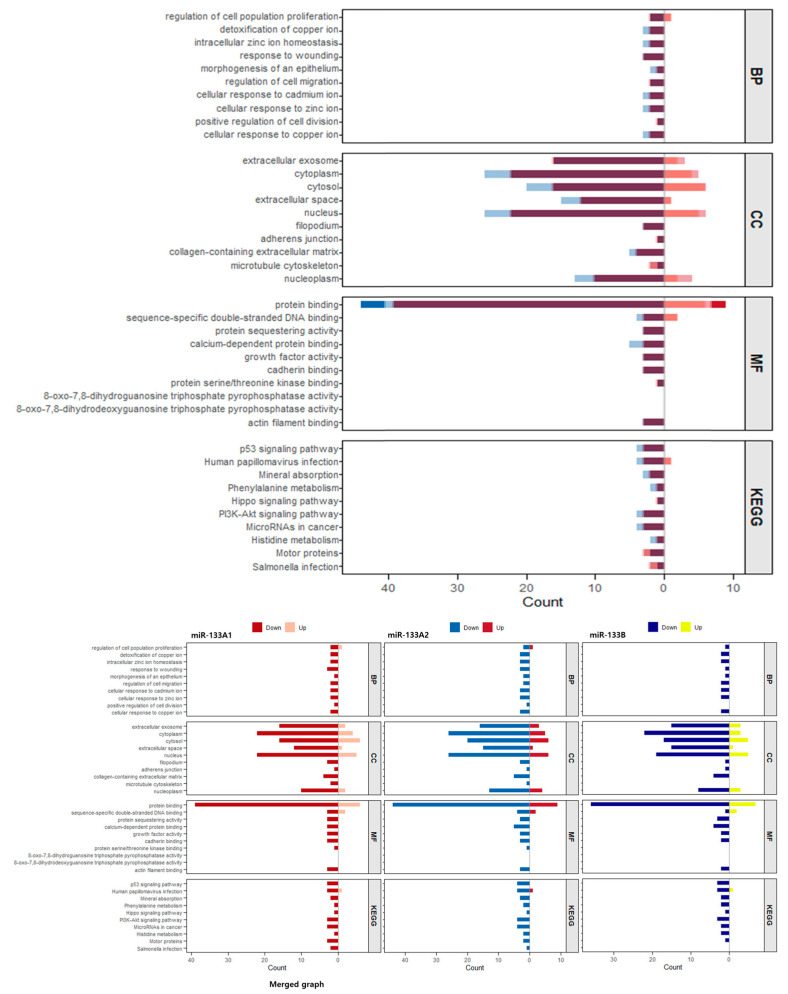
Comparative GO and KEGG enrichment analysis of differentially expressed genes across *miR-133* isoforms. The upper panel presents a merged visualization of enriched GO categories (BP: Biological Process, CC: Cellular Component, MF: Molecular Function) combining data from all three *miR-133* isoforms in a single integrated bar plot. The lower panel displays KEGG pathway enrichment analysis. Bar plots show enrichment in SW48-KI*133A1* (red/pink bars), SW48-KI*133A2* (blue/light blue bars), and SW48-KI*133B* (dark blue/yellow bars) compared with parental SW48 controls. For each isoform, darker-colored bars represent downregulated gene sets and lighter-colored bars represent upregulated gene sets. Bars are grouped by functional category to facilitate direct comparison across the three isoforms. The side-by-side presentation reveals highly convergent enrichment patterns across all three *miR-133* isoforms, with consistent regulation of cell proliferation, metal ion homeostasis, extracellular components, protein binding functions, and cancer-associated signaling pathways.

To examine potential isoform-specific differences, GO and KEGG enrichment analyses were performed separately for upregulated and downregulated gene sets in each *miR-133* isoform (Figure 5). The side-by-side comparison reveals that all three isoforms display highly similar enrichment patterns, indicating a predominantly shared core regulatory program. Across all isoforms, downregulated genes were consistently enriched in biological processes related to regulation of cell population proliferation, detoxification of copper ion, intracellular zinc ion homeostasis, and cellular responses to metal ions. In the cellular component category, enrichment in extracellular exosome, extracellular space, and cytoplasm was observed consistently across isoforms, with subtle quantitative differences in the magnitude of enrichment. Molecular function analysis showed consistent regulation of protein binding-related activities, including calcium-dependent protein binding and protein sequestering activity, across all three isoforms. KEGG pathway analysis revealed that *miR-133A1*, *miR-133A2*, and *miR-133B* commonly modulate cancer-associated signaling pathways including p53 signaling pathway, PI3K-Akt signaling pathway, and Hippo signaling pathway, with highly comparable enrichment patterns. While minor quantitative variations in enrichment scores were observed between isoforms, the overall functional landscape remained remarkably consistent, suggesting that the three *miR-133* isoforms predominantly regulate overlapping biological processes through convergent mechanisms, with only subtle isoform-specific nuances in the magnitude of pathway modulation.

### 3.6. Isoform-Specific Differential Expression of Representative Genes

**Figure 6:**
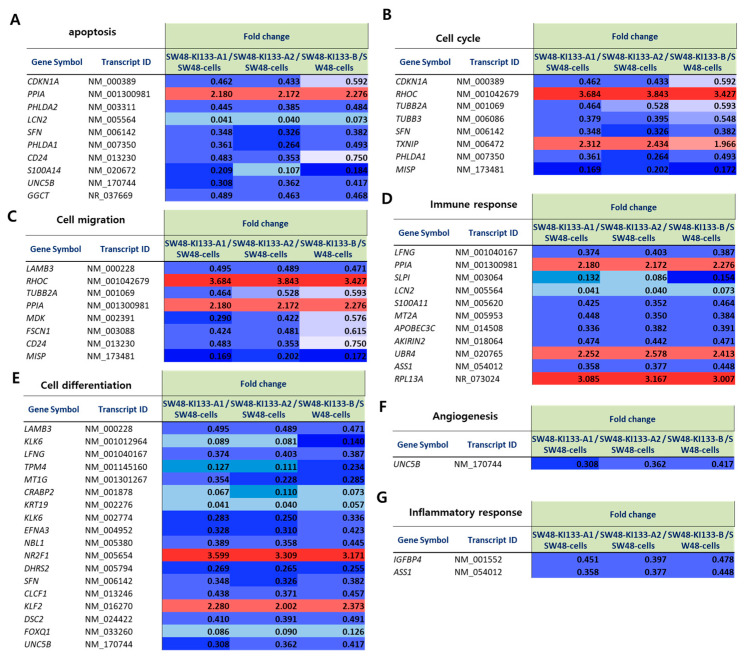
Heatmap representation of fold change values for representative genes regulated by *miR-133* isoforms. Heatmaps show the relative expression (fold change) of selected genes in SW48-KI*133-A1*, SW48-KI*133-A2*, and SW48-KI*133-B* compared with parental SW48 controls. Red indicates upregulation (fold change > 1.5), and blue indicates downregulation (fold change < 0.67). (**A**) Apoptosis-related genes. (**B**) Cell cycle genes. (**C**) Cell migration genes. (**D**) Immune response genes. (**E**) Cell differentiation genes. (**F**) Angiogenesis-related gene. (**G**) Inflammatory response genes. Note that some panels share common genes (e.g., PPIA) as these genes are functionally involved in multiple biological processes.

To validate the transcriptomic alterations induced by *miR-133* isoforms, we analyzed representative sets of differentially expressed genes and visualized them by heatmap (Figure 6A–G). Apoptosis-related genes were consistently downregulated across all isoforms, with *PPIA* showing upregulation (Figure 6A). Cell cycle regulators displayed similar patterns to apoptosis genes, with *RHOC* and *PPIA* markedly upregulated while most other genes showed downregulation (Figure 6B). Genes involved in cell migration exhibited mixed regulatory patterns, with *RHOC*, *TUBB2A*, and *PPIA* upregulated while others such as *LAMB3*, *MDK*, and *FSCN1* were downregulated (Figure 6C). Immune response-related genes displayed diverse expression patterns, with *PPIA* upregulated and *UBR4* and *RPL13A* showing particularly strong upregulation, while genes such as *LFNG*, *LCN2*, and *ASS1* were downregulated (Figure 6D). In cell differentiation pathways, most genes including *LFNG*, *TPM4*, and *MT1G* were downregulated, with notable exceptions including *NR2F1* and *KLF2*, which showed marked upregulation (Figure 6E). Angiogenesis-related gene *UNC5B* was consistently downregulated across all three isoforms (Figure 6F). Inflammatory response genes *IGFBP4* and *ASS1* were downregulated (Figure 6G). Together, these results demonstrate that *miR-133* isoforms orchestrate transcriptional programs affecting multiple cellular processes, with both shared and isoform-specific regulatory patterns.

## 4. Discussion

MicroRNAs have long been recognized as central regulators of tumor biology, with *miR-133* frequently described as a tumor suppressor across multiple cancer types including gastric, lung, and breast cancers [10,11,12,19]. Previous reports have highlighted its role in inhibiting cell proliferation and promoting apoptosis, primarily by targeting oncogenic regulators such as *EGFR, FSCN1*, and *MET* [20,21]. In colorectal cancer (CRC) specifically, *miR-133* has been reported to target multiple oncogenic factors including *LASP1* [22], *TAGLN2* [13], *FSCN1* [23], and *COL1A1* [24], thereby suppressing proliferation, migration, and invasion. However, most studies have treated *miR-133* as a single entity, and the functional differences between its isoforms have remained largely unexplored [25]. Our findings extend this knowledge by demonstrating that *miR-133* isoforms, while sharing a conserved tumor-suppressive program, exert highly convergent transcriptional effects with subtle, isoform-specific differences that may significantly contribute to CRC heterogeneity (Figure 2, Figure 3, Figure 4, Figure 5 and Figure 6).

Our transcriptomic analysis revealed that all three *miR-133* isoforms regulate highly overlapping biological processes. Genes involved in apoptosis, cell cycle, cell migration, immune response, cell differentiation, angiogenesis, and inflammatory response showed consistent regulatory patterns across isoforms (Figure 6A–G). Functional enrichment analysis further confirmed this convergence, with all three isoforms similarly affecting proliferation, metal ion homeostasis, extracellular components, and major cancer-associated signaling pathways including p53, PI3K-Akt, and Hippo signaling (Figure 4 and Figure 5). This shared regulatory program suggests that the core tumor-suppressive or oncogenic functions of *miR-133* are largely conserved across isoforms.

Despite this overall similarity, subtle quantitative differences were observed. Hierarchical clustering analysis showed that while KI*133A1* and KI*133A2* clustered more closely at lower fold change thresholds, KI*133B* exhibited a slightly more distinct transcriptional profile (Figure 3A). At stringent thresholds (fold change >3.0), core regulatory genes such as *ADGRL2, NR2F1, S100A16*, and *KRT19* showed differential expression magnitudes across isoforms, suggesting that isoform-specific fine-tuning of key genes may occur even within a largely overlapping regulatory framework. Additionally, pathway enrichment analysis revealed marginal differences in the extent of proliferation pathway suppression and cancer-associated pathway upregulation among isoforms (Figure 5), with KI*133B* showing slightly more prominent upregulation of certain signaling pathways.

Our findings reveal predominantly convergent functional landscape, with quantitative variation among *miR-133* isoforms despite near-identical mature sequences. While *miR-133A1* and *miR-133A2* are identical and share an identical seed region with *miR-133B* (differing only at position 22: G→A), distinct transcriptomic profiles were observed across the three isoforms. This paradox may be explained by non-canonical mechanisms including 3′-supplementary pairing contributions to target specificity, differential pre-miRNA processing efficiency and stability, RNA-binding protein recruitment, and potential contributions of co-encoded passenger strands (miR*). While miR-133A* is well characterized, our RNA-seq dataset detected only minimal reads (<5 per million) corresponding to miR-133B*, suggesting that it may be expressed at very low or condition-dependent levels. Given that murine miR-133B* has been reported in small RNA sequencing studies, a conserved but weakly expressed human homolog cannot be ruled out. We have therefore revised the text to emphasize that this mechanism remains a plausible contributor to isoform-specific effects. Future AGO-CLIP and strand-specific small RNA-seq analyses will be essential to determine whether miR-133B* is functionally active in human cells.

Clinical relevance is also an important consideration. Publicly available colorectal cancer datasets (e.g., TCGA and GEO) demonstrate that *miR-133* isoforms are frequently downregulated in tumor tissues compared with adjacent normal mucosa, and lower expression has been associated with advanced stage and poor prognosis [26,27,28]. These clinical observations are consistent with multiple reports showing that *miR-133* downregulation correlates with increased tumor size, lymph node metastasis, and reduced overall survival in CRC patients [29]. Furthermore, restoration of *miR-133* expression has been shown to sensitize CRC cells to chemotherapy and inhibit tumor growth in xenograft models [30], supporting its potential as a therapeutic target. Although our present study focused on an in vitro model, these findings suggest that isoform-specific regulation of *miR-133* may have translational relevance. Future studies incorporating patient-derived samples and correlating isoform levels with clinical outcomes will be essential to validate their potential as biomarkers or therapeutic targets.

While our transcriptomic study provides important insights into isoform-specific regulation by *miR-133*, several limitations should be acknowledged. First, this study focused solely on overexpression models. Complementary knockdown experiments, using antagomiRs or CRISPR interference, will be essential to validate the dependency of colorectal cancer cells on endogenous *miR-133* activity and are planned for future work. Second, although *miR-133A1* and *A2* encode identical mature sequences [31], differences in promoter activity, precursor stem-loop structure, and local chromatin context may result in distinct transcriptional regulation and processing efficiency. In addition, *miR-133B* differs from A1/A2 by a single nucleotide at the 3′ end, which, although outside the canonical seed region, may influence thermodynamic stability, Argonaute loading, or target site pairing at the 3′ compensatory region. Such post-transcriptional differences could explain the subtle but reproducible divergence observed in our transcriptomic profiles. The relatively modest overlap among dysregulated genes further suggests that many observed changes may be indirect or secondary effects [32,33]. Importantly, the transcriptomic trends described here were reproducible across biological replicates, suggesting that these isoform-specific effects, while subtle, represent consistent regulatory phenomena rather than experimental noise. Third, our analysis was limited to a single colorectal cancer cell line (SW48). While this model provided a controlled system to dissect isoform-specific responses, it cannot fully capture the heterogeneity of CRC. Validation across additional CRC cell lines and patient-derived tissues will therefore be critical to generalize our findings. Fourth, although lentiviral overexpression ensured robust induction of each isoform [34], it may not accurately reflect endogenous expression ratios or regulatory dynamics. This artificial modulation could bias transcriptomic outcomes. Future studies employing CRISPR-based modulation, inducible expression systems, or isoform-specific antagomiRs will be important to approximate physiological regulation and validate biological relevance.

In conclusion, our study demonstrates that *miR-133* isoforms exert highly convergent transcriptional effects in colorectal cancer cells, regulating overlapping pathways related to proliferation, apoptosis, migration, and major cancer-associated signaling cascades. While subtle quantitative differences exist, the overall functional landscape is remarkably consistent across isoforms. These findings suggest that the biological effects of *miR-133* are primarily determined by the shared seed sequence and core target repertoire, with isoform-specific variations playing a relatively minor modulatory role. Future mechanistic studies and clinical validation will be important to fully understand the therapeutic implications of *miR-133* regulation in colorectal cancer.

## Data Availability

The raw data supporting the conclusions of this article will be made available by the authors on request.

## Abbreviations

The following abbreviations are used in this manuscript:

CRC	Colorectal cancer
miRNA	MicroRNA
DEG	Differentially expressed gene
GO	Gene Ontology
KEGG	Kyoto Encyclopedia of Genes and Genomes
BP	Biological Process
CC	Cellular Component
MF	Molecular Function
ECM	Extracellular matrix
FDR	False discovery rate
RIN	RNA integrity number
RNA-seq	RNA sequencing
